# Understanding Spatter and Its Spatial Variations in Laser Powder Bed Fusion of Ti6Al4V

**DOI:** 10.3390/ma19214575

**Published:** 2026-10-29

**Authors:** Daniel P. Lund, Mark Sorna, Dwayne D. Arola, Mamidala Ramulu

**Affiliations:** 1 Department of Materials Science and Engineering, University of Washington, Seattle, WA 98195, USA; dplund@uw.edu (D.P.L.); mksrna@yahoo.com (M.S.); 2 Department of Mechanical Engineering, University of Washington, Seattle, WA 98195, USA

**Keywords:** computer vision, in situ data, laser powder bed fusion, spatter

## Abstract

**Highlights:**

- Spatter is evaluated via optical tomography (OT) in situ imaging acquired during builds performed with Ti6Al4V powder.
- The volume of generated spatter appears higher in upwind regions of the build chamber than downwind regions.
- Interaction between spatter and the inert gas flow contributes to spatial variations in the volume of spatter generated.
- Spatial variations in the inert gas flow are estimated from the trajectories of the spatter particles observed in OT data.
- More spatter is generated when printing with gas atomized powder compared to plasma atomized powder.

**Abstract:**

Additive manufacturing (AM) by laser powder bed fusion (L-PBF) is rapidly developing as a principal method for the manufacture of metal parts. However, widespread adoption of the technology, especially for safety-critical applications, is impeded by complications to achieving the desired component quality and reliability. Spatter occurs due to interactions between the laser and the melt pool and involves the ejection of material from within or about its vicinity. Recognized as a process signature in L-PBF, spatter is considered detrimental to build quality. However, relationships between spatter kinetics and part defects have remained largely underexplored. In this investigation, spatter is evaluated via in situ optical tomography (OT) imaging acquired during builds performed with Ti6Al4V powder. Results showed that the volume of spatter content is greater at particular scan angles as well as specific locations in the build chamber. Furthermore, the spatter dynamics behave differently upwind and downwind of printed regions and indicate that local variations in gas flow can be inferred from spatter signatures. As spatter is known to directly cause defects in parts produced by L-PBF, regions with increased spatter potentially signal reduced metal quality. Variations in the spatter content are identified and regions of the build at greater risk for defects are highlighted.

## 1. Introduction

In response to the constraints of traditional methods of manufacturing and the impressive advancements in commercial technology for laser powder bed fusion (L-PBF), applications for this technology have grown rapidly in recent years. Widespread adoption of additive manufacturing (AM) methods is, however, limited by the difficulty in controlling process variation and its contribution to part quality [1]. This concern is paramount in L-PBF of stress-critical and safety-critical components [2,3,4,5]. Variability in metal quality can interfere with the certification process [6] and increase the cost of qualification [7].

**Figure 1:**
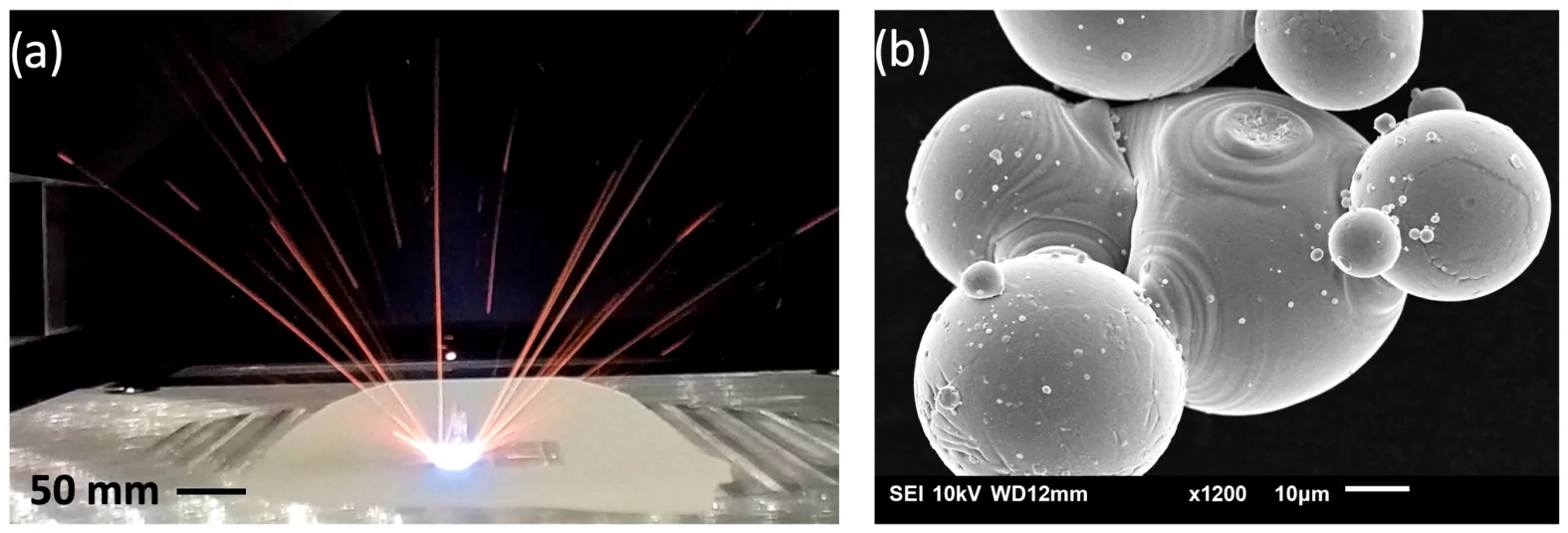
Spatter characteristics in L-PBF of Ti6Al4V. (**a**) Emission of spatter from the powder bed indicated by the emitted sparks, and (**b**) representative spatter particle consisting of an agglomeration of powder particles and molten metal ejected from the melt pool.

Spatter is a process phenomenon in L-PBF [8,9] and refers to the ejection of material from within or about the vicinity of the melt pool that occurs due to laser–material interactions [10]. Spatter can be composed of molten material ejected from the melt pool or partially melted agglomerations and unmelted powder particles entrained by the vapor jet evolved from the melt pool [10,11]. Examples of spatter emission in L-PBF of Ti6Al4V and the morphology of resulting spatter particles are shown in Figure 1a, and Figure 1b, respectively. Because spatter particles are typically larger than the powder particles [12], they can be more difficult to melt, resulting in lack-of-fusion (LOF) defects [8]. Spatter particles can also re-enter the path of the laser beam and disrupt the transmission of laser energy to the powder bed [13,14], which can cause the development of LOF defects [13,15]. Moreover, the undesired thermal energy delivered to airborne material can further influence byproduct evolution [10,11].

Spatter that is redeposited onto the surface of the build results in protrusions that can disturb the recoater blade, causing local fluctuations in the powder layer thickness [10]. While it may not be possible to produce L-PBF parts without generating spatter [16], its emission can be minimized by managing process variables [13,17,18] and powder condition [19,20]. Spatter is influenced by a number of attributes of the L-PBF process, and a brief overview of current research in relevant areas is presented in the following section.

## 2. Background

One of the primary concerns regarding components produced by metal AM is spatial variation in metal performance, i.e., that components printed in one location within the build space exhibit properties unique from those that are printed in another location [1,6,10,21]. The premise here is that the generation of spatter could contribute to the development of anomalies that contribute to metal performance. Moreover, characterizing spatial variations in spatter emission could be used as a diagnostic tool to infer metal quality and assist in quality control.

Important advancements have been made in understanding spatter dynamics and spatial variations in L-PBF through simulations. Ly et al. [22] performed multiphysics simulations of the L-PBF process to observe the mechanics of particle denudation. The investigation exposed that a low-pressure region surrounding the upward-driven metal vapor jet draws in nearby metal particles, which are then launched upward and deposited elsewhere on the build surface [22]. The radius of this low-pressure region surrounding the laser beam is approximately 10x that of the laser [23], and the high temperature of the plume contributes to particle agglomeration [23]. Three unique types of spatter were observed, including i) cold entrainments, where denuded particles miss the laser beam and metal vapor jet, ii) hot entrainments, where particles are heated by the laser beam and/or the metal vapor plume, and iii) recoil pressure-driven ejections, where molten metal is ejected from the melt pool by metal vapor pressure. A similar simulation was performed in Matthews et al. [11], which identified that an additional mechanism of denudation arises due to the melt pool itself, entraining nearby unmelted powder particles through capillary forces. However, the range of this effect is limited to the diameter of the powder particles. In contrast, the range of vapor jet entrainment was estimated by Ly et al. be approximately two melt pool widths [22]. Nevertheless, only about 15% of the observed spatter originates from the melt pool and approximately 60% is generated by hot entrainments [22].

The spatter behavior observed in simulations has been confirmed through high-speed in situ imaging. In general, physical observations indicate that spatter particles are larger than the mean particle size of the feedstock powder [24]. The distribution of redeposited spatter can be approximated by identifying spatter features in high-resolution imaging and calibrating these with the actual spatter particles in the retrieved powder volume as determined through microscopy [25]. Oversized spatter particles have been observed to originate from stochastic collisions between particles ejected nearly simultaneously from the melt pool, as well as particles ejected from distant locations [24]. The heat flow from the vapor plume also contributes to the coalescence of partially sintered powder agglomerates [24]. Interestingly, the orientation of the vapor jet can change from leading to following the direction of laser scanning, which largely depends on laser power and scan speed [23]. Utilizing the higher resolution of high-speed X-ray imaging, the phase changes of powder from solid to liquid and liquid to gas can be observed. Using this approach, Guo et al. determined that the driving force of particle acceleration from vapor pressure is approximately an order of magnitude higher than the force of the inert gas flow [26]. That suggests that spatter particles will be hard to steer by gas flow alone.

Very recent work using high-speed imaging has shown that the motion of spatter and denuded powder particles can be monitored with an off-axis mounted camera [27,28] and used to estimate layerwise print quality. Furthermore, the motion of spatter particles can be tracked in 3D using plenoptic [29] or stereo [30] high-speed imaging methods. These higher-resolution approaches to tracking of spatter have highlighted the importance of this topic and the need to mitigate the detrimental effects on build quality.

Are there strategies for reducing spatter emission? One suggestion involves pre-sintering the powder bed to reduce the amount of spatter generated [26], an approach confirmed to be effective in a very novel study reported by Khairallah et al. [19]. The volume of denuded powder, and ultimately the volume of spatter generated, can also be reduced by increasing the environmental pressure of the build chamber [11,26].

It is widely believed that the inert gas flow distribution is key to effective removal of spatter and other process byproducts [13,31] and that the gas flow distribution ultimately influences the quality of the built metal [10,20]. A recent experimental study identified spatial variations in the flow distribution within the build chamber of a commercial L-PBF system, as well as regions of stagnation that allow process byproducts to accumulate [32]. Selective improvements to the ductwork that supplies the inert gas have been shown to improve the gas flow distribution [32], providing a path for further improvements.

Are engineered changes in gas flow sufficient to control spatter? A coupled computational fluid dynamics/discrete element modeling (CFD-DEM) simulation was used to simulate the motion and redeposition locations of spatter particles [33]. As expected, the results confirmed that the inert gas flow distribution has a significant influence on the spatial distribution of redeposited spatter particles [33]. Experiments performed by Baehr et al. [34] evaluated the effectiveness of an argon–helium mixture at removing spatter. Under the appropriate flow conditions, the mixture provided the same effectiveness at removing spatter and condensate from the build chamber as pure argon. But, more importantly, this mixture reduced the total amount of spatter generated, which is likely due to improved melt pool cooling from the use of helium [34]. Zhang et al. used CFD to show that the Coanda effect is the dominant mechanism limiting the removal of spatter from the build chamber; they demonstrated that the “clear rate” can be improved from 70% up to 95% by elongating the inlet nozzles [35]. The change in gas flow distribution obtained through simulation was confirmed experimentally using hot wire anemometry on a commercial L-PBF system [35], which demonstrated the importance of optimizing processing conditions in L-PBF.

Due to the value of the data acquired, in situ imaging systems have advanced from custom systems constructed by pioneering end-users (e.g., [17]) to complete toolboxes offered as standard operating equipment on many commercial AM systems. Melt pool data has already received significant attention due to observed relationships between melt pool temperature fluctuations and metal porosity. However, the focus on spatter data has rightfully increased, which can provide better representation of anomalous events and in turn support models that provide more effective prediction of anomalies in the printing process [36]. A summary of the research efforts relevant to in situ monitoring of spatter and related phenomena is listed in Table 1.

While monitoring spatter using in situ data appears promising to forecast or infer build quality, no study has evaluated the spatial variations in generated spatter and spatter kinetics and their relationship to build configuration, processing conditions, and metal quality. And while OT data has received significant attention towards identifying local anomalies in printed metal, to the authors’ knowledge it has not been employed to evaluate the dynamic characteristics of spatter. Therefore, the primary objectives of this study are to characterize spatial variations in spatter across the build space utilizing in situ OT data, to identify features representative of gas flow characteristics, and to identify build variables that influence spatter dynamics. Lessons learned are then applied to interpret potential relationships between spatter characteristics observed in situ and metal quality.

## 3. Materials and Methods

### 3.1. Materials and Printer

The builds in this investigation were conducted using two different titanium powders having nominal composition of Ti6Al4V, including a gas atomized (GA) powder, AncorTi Ti6Al4V (GKN, Cinnaminson, NJ, USA), and a plasma atomized (PA) powder, EOS Titanium Ti64 (EOS GmbH, Turku, Finland). The PA powder from EOS meets specifications of ASTM F2924 [39], and the GA powder from GKN was manufactured in accordance with standards defined by AS9100D [40]. The particle size distribution (PSD) was measured using laser diffraction and is listed in Table 2.

The builds were performed using an EOS M290 (EOS GmbH, Krailling, Germany) which utilizes a 400 W ytterbium fiber laser with a wavelength of 1060–1100 nm and features a build area of 250 × 250 mm square and height of 325 mm. All specimens were printed using the “Ti64_SpeedM291 1.10” parameters per the details listed in Table 3 [41]. The laser scan angle for the first layer is 67° and increments by 67° for each subsequent layer. Argon was used as the shielding gas for all builds and the machine was configured with a grid nozzle and steel recoater blade. The machine was equipped with EOSTATE, (EOS GmbH, Krailling, Germany) an in situ suite for system monitoring that includes melt pool imaging, OT imaging, visible light imaging of the post-exposure and post-recoat powder bed surface, as well as monitoring of system variables [42]. The latter includes axis positions, temperature, differential pressure in the gas flow recirculation system, and oxygen level in the build chamber.

### 3.2. Build Design

**Figure 2:**
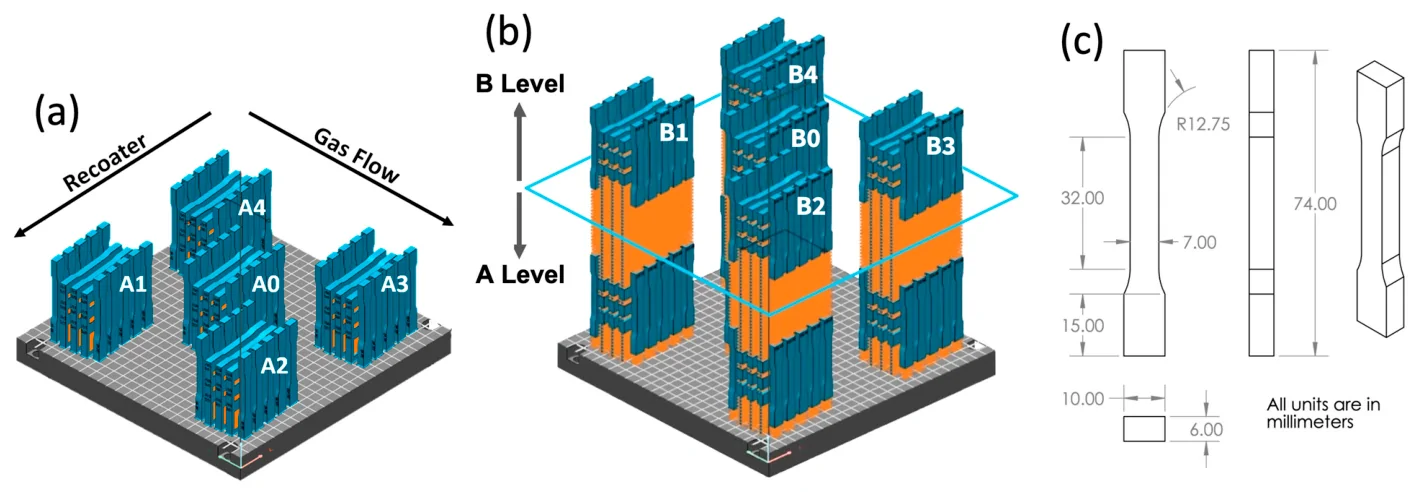
Details of the build design outlining the array of tensile coupons with vertical and horizontal orientations for the builds performed with (**a**) gas atomized and (**b**) plasma atomized powders. Labels indicate the build zones (0 to 4) on two levels (A and B). Blue indicates dense metal and orange indicates support structure. (**c**) Tensile specimen geometry.

The build design consisted of a cluster of tensile specimens distributed across five different zones and two different levels, as shown in Figure 2. The build is part of a larger effort consisting of a round robin investigation that is focused on property variability in L-PBF [21]. The specimens were grouped in clusters within the 5 zones of the build chamber. Zone 0 is at the center, and Zones 1 to 4 occupy each of the four corners of the build space as shown in Figure 2a. The cluster of specimens in each zone consisted of vertical and horizontal orientations. The coupon orientations were rotated in the build plane 5° counter-clockwise from the *x*-axis to minimize disruption of the recoater blade as it passes over the printed regions. Each cluster consisted of 19 tensile specimens (10 vertical and 9 horizontal) that conformed to the subsize dimensions outlined by ASTM E8 [43] as shown in Figure 2b.

To support the effort focused on variability in mechanical properties, multiple builds were performed in the present study and printed with either the PA or GA powder. The build performed with the GA powder is shown in Figure 2a and consisted of a total of 95 specimens, including 50 vertical and 45 horizontal specimens. Due to constraints in the volume of powder available from that experimental lot, specimens were printed in the A level only with the GA powder. The build performed with PA powder is shown in Figure 2b and included a full complement of specimens in each of the five zones on both the A and B levels for a total of 100 vertical and 90 horizontal specimens, respectively, and 190 specimens per build.

For each powder, Build 1 was performed with virgin powder, and Builds 2 to 6 were performed with a blend of recovered powder from the previous build and virgin powder added to maintain consistent volume. Prior to the addition of virgin powder, large spatter particles and agglomerates were removed from the recovered powder with a 63 µm sieve. The powder chemistry and particle size distribution were monitored to ensure there were no adverse changes over the number of reuse cycles [38].

### 3.3. Data Collection

The EOSTATE in situ monitoring suite in stock OEM configuration was used to capture optical tomography (OT) images of the entire build area in each layer throughout the build and for all builds performed with the GA and PA powders. Images were captured using a 5-megapixel sCMOS camera (Excelitas PCO GmbH, Kelheim, Germany) mounted in the top of the build chamber. This camera captures temporally integrated images with a rolling shutter at a frequency of 10 Hz, and the resulting images are integrated to produce a single image per layer [44]. The portions of metal that are heated by the laser beam exhibit radiant emissions, and the images capture a time-integrated representation of infrared (900 ± 25 nm) radiation emitted within the build area. This radiation may originate from different forms of material, such as exposed regions, heated powder particles, and molten metal. The OT images were saved as 16-bit grayscale images with a resolution of 2000 by 2000 pixels, which results in a spatial resolution of 125 µm.

**Figure 3:**
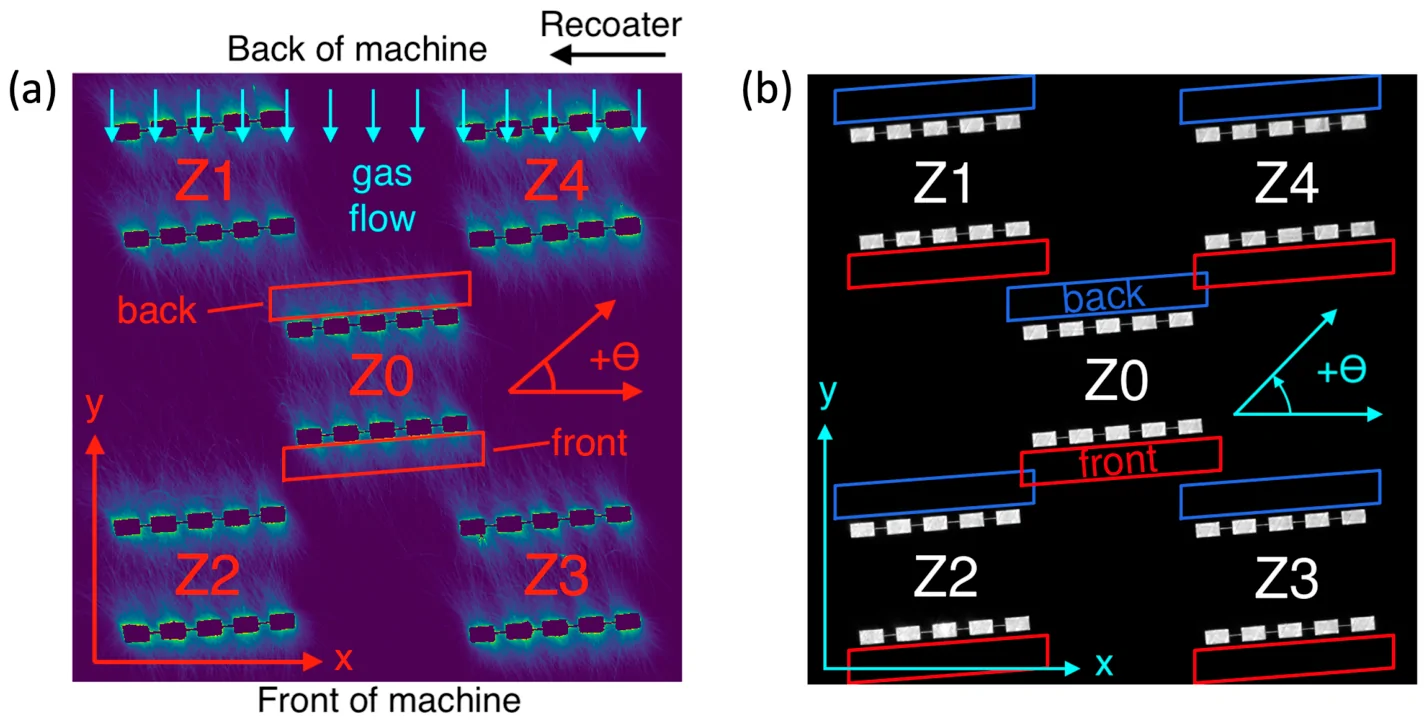
Definitions used in evaluating the OT images. (**a**) In situ obtained OT image of a layer, labeled to define analysis regions and variables. (**b**) Regions Of Interest (ROIs) identified within the build space. Front zones shown in red, back zones shown in blue.

The spatter distribution was evaluated over the entire build area with respect to machine coordinates as shown for the OT image of a selected layer in Figure 3a. In this figure, the printed regions have been masked to prevent grayscale saturation and emphasize spatter features. The coordinate system used in this analysis is shown at lower left in each image, along with the counter-clockwise definition of angles with respect to the positive x-axis, as shown at center right in each image. The zones are labeled for clarity.

**Figure 4:**
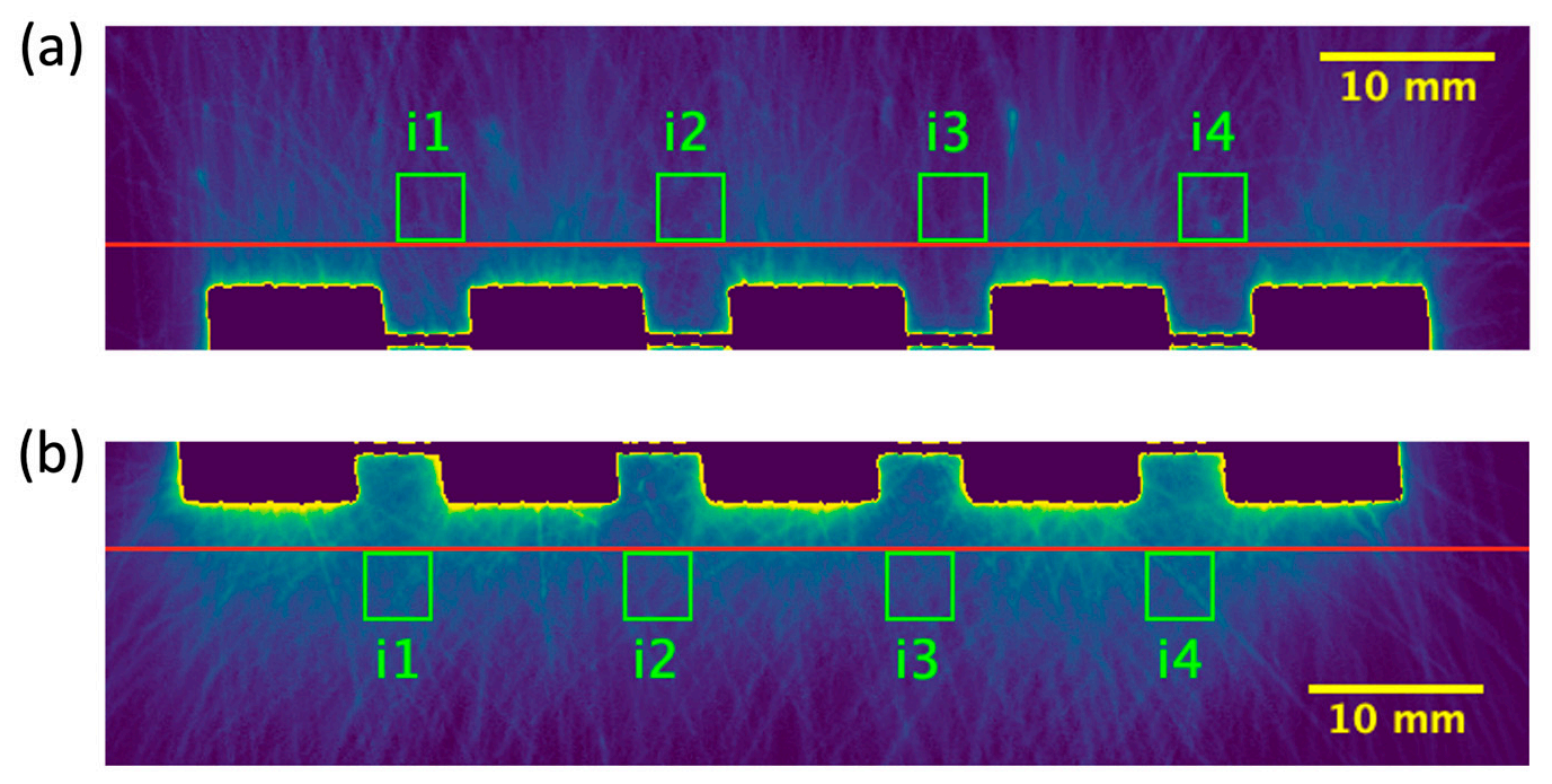
Definitions of ROIs used for grayscale evaluation. (**a**) Identification of inter-specimen regions (ISR) for the (**a**) back and (**b**) front ROIs of Zone 0. The areas below the red line in (**a**) and above the red line in (**b**) are not part of the ROI but included for reference to specimen locations.

For each zone, a region of interest (ROI) was defined in front and behind the cluster of specimens as shown in Figure 3b, producing 10 ROIs for each layer. The sectioned ROIs were reshaped from parallelograms into rectangles by shifting the left and right edges of the parallelograms to be in vertical alignment with each other. Examples of the transformed ROIs are shown in Figure 4a,b. Note the identification of inter-specimen regions (ISR) i1 to i4 in the transformed ROIs, each in equal proximity to the printed regions.

All the images examined in this study were from layers within sections of the build where only the grip sections of the vertical specimens were being printed. Doing so allowed examination of spatter in immediate proximity to the vertical specimens from which it was generated, without the influence of spatter from nearby horizontal specimens in the same cluster.

#### 3.3.1. Area Intensity

The OT images exhibit an inherent spatial variation in grayscale intensity related to the distance from the camera, with features near the center of the build plate exhibiting higher grayscale levels than features near the corners. To remove this background intensity, a second-order polynomial fit was used to spatially normalize all OT images and reduce the influence of this variation.

Figure 4a presents the four inter-specimen regions (ISRs) for one of the ROIs, each being defined as a 3.75 mm square. There are a couple of advantages of defining the ISRs in this manner. Positioning the ISRs in locations not immediately adjacent to the specimens avoids saturation of grayscale levels and makes variations in grayscale more apparent. Also, defining the ISRs next to the gaps in between the specimens emphasizes the influence of ejection forces. Gas flow acts (primarily) parallel to the *y*-axis (Figure 3), and observation of spatter in the ISRs indicates a component of motion parallel to the *x*-axis, which cannot be driven by gas flow alone.

After applying the correction for the distance from camera variation in grayscale intensity, the OT images were sectioned into 10 ROIs and the ROIs reshaped from parallelograms into rectangles as mentioned earlier. The average grayscale intensity across the 4 ISRs as shown in Figure 4a,b was then evaluated for each of the 10 ROIs in a layer, producing an average inter-specimen grayscale measurement for each ROI. By repeating the evaluation of inter-specimen grayscale intensity for a series of layers, each with a different scan angle, a grayscale intensity distribution was constructed as a function of scan angle. This analysis was conducted for the builds performed with both the PA and the GA powders.

#### 3.3.2. Spatter Trajectories

The orientation of spatter features was evaluated using the OrientationJ plugin (Biomedical Imaging Group, EPFL University, Lausanne, Switzerland, version 2.0.7) for ImageJ (National Institutes of Health, version 2.14.0/1.54f). The dominant direction of spatter was determined for each of the 10 ROIs in a sample of 24 sequential OT images.

**Figure 5:**
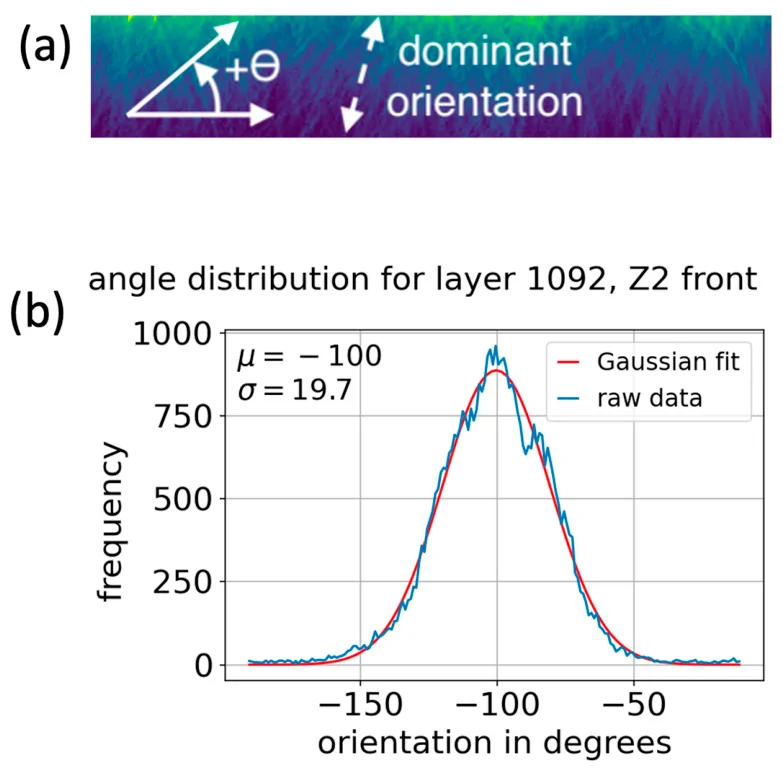
Details for post-processing of the OT images for spatter characteristics. (**a**) Representative ROI image, indicating dominant direction of spatter. (**b**) Corresponding histogram for orientation in a spatter ROI with curve fit.

An example ROI used for evaluating spatter angle trajectories is shown in Figure 5a, and a histogram for the orientations of the spatter features observed in this ROI is presented in Figure 5b. The domain of the histogram was rotated such that the peak of the histogram appears in the center of the domain, which enabled fitting a Gaussian distribution to the data. As evident in the histogram in Figure 5b, the mean angle of spatter ray for this ROI is −100° and the standard deviation is 19.7°.

#### 3.3.3. Tensile Testing

To assess the quasi-static properties and compare these properties to metrics of spatter, a subset of the total number of built coupons produced with PA powder were evaluated by tensile testing according to standard methods defined by ASTM E8 [21]. Prior to performing the testing, the specimens were subjected to a stress-relief annealing heat treatment, followed by machining of all surfaces of the gauge section to conform to the standard [21]. The heat treatment cycle was performed in an argon atmosphere furnace (Nabertherm, model LH 120; Litienthal, Germany) and consisted of a one-hour ramp to 745 °C, a two-hour soak at that temperature, followed by furnace cooling to room temperature [21]. A rapid air cooling was allowed once the temperature of the furnace dropped below 500 °C, below which no microstructural changes were expected [21]. Following heat treatment, all surfaces of the gauge section were machined with a minimum of 0.5 mm of material removal, which exceeds the minimum dictated by AMS2801B [45] for a similar heat treatment performed in ambient air [21]. Tensile testing of the specimens was performed on a commercial universal testing system (Instron, model 5585H; Norwood, MA, USA) at 1.27 mm/min for a 25 mm gauge section length [43]. Displacement was measured with a clip-on extensometer (Instron, Norwood, MA, USA) and used to quantify the strain response. The yield and ultimate tensile strength and the strain at failure were evaluated from the resulting data.

For each zone in Builds 1 and 6 performed with PA powder and across Levels A and B, four vertical coupons were chosen pseudo-randomly, culminating in approximately 80 vertical coupons. Specifically, two vertical specimens were randomly selected from the back row (specimens 1 to 5 adjacent to the back ROI as seen in Figure 3) and front row (specimens 6 to 10). Builds 1 and 6 each had a different selection of coupons that were tested. Although most tensile tests were successful, a few were not due to grip slip or fracture outside the instrumented section of the gauge section [21]. This reduced the number of successful tests from 80 to 75 coupons.

The specimens subject to tensile testing were sorted according to build number (Builds 1 and 6), build level (Level A or Level B), and ROI (back or front), for a total of 2 × 2 × 2 = 8 groups. The values for strain at failure for each specimen were compared to the corresponding average grayscale intensity of the specimen spatter region (SSR) adjacent to that specimen. The data generated from this analysis was then plotted to assess potential relationships between the strain at failure and spatter intensity.

## 4. Results

### 4.1. Direction of Spatter Trajectories

**Figure 6:**
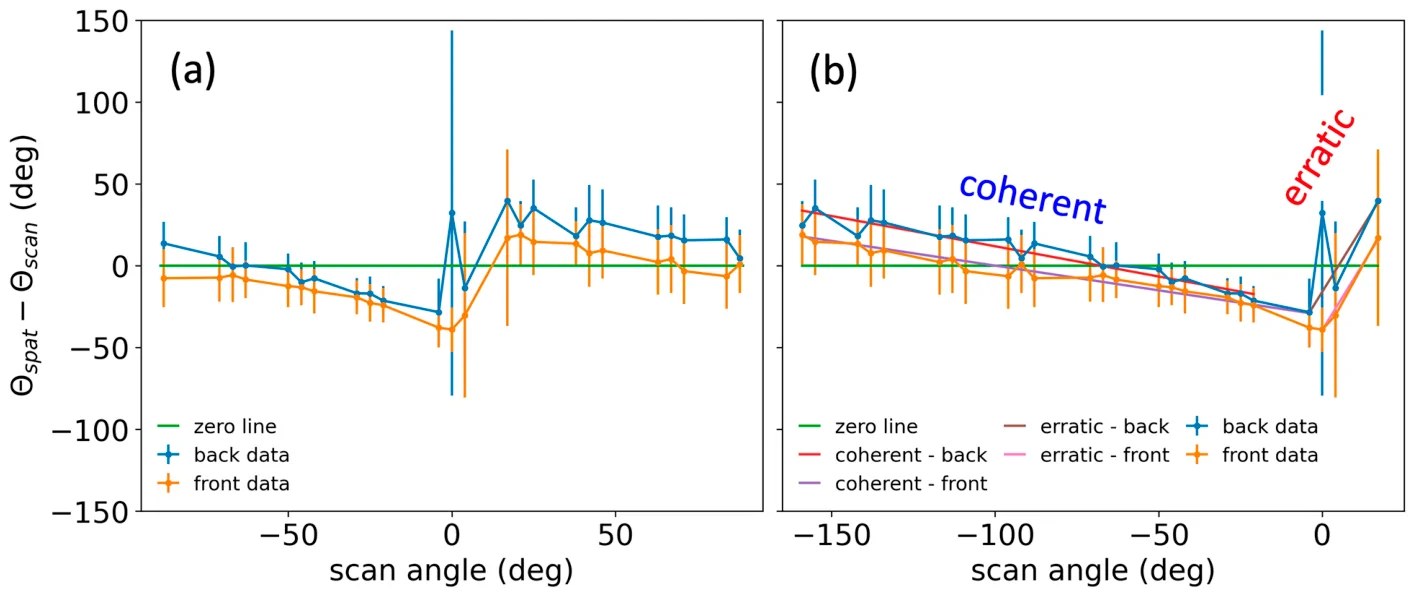
Spatter emission characteristics. (**a**) Peak spatter angle difference from scan angle for Zone 0 only. (**b**) Linear fit for the direction of the spatter trajectories in Zone 0 in terms of the scan angle data. The domain of the data has been rotated so the linear regions can be presented uninterrupted. This data includes the spatter in the back and front ROIs.

The difference between the spatter angle with maximum intensity and the scan angle is plotted as a function of the scan angle in Figure 6a. Points near the *x*-axis indicate that the spatter angle is effectively coincident with the scan angle, whereas points above or below the *x*-axis indicate that the spatter angle is greater than or less than the scan angle, respectively. In Figure 6b, the domain of the data has been rotated to emphasize two distinct regions of spatter–scan angle response. There is a stable section that spans most of the domain with cohesive data points and low to moderate uncertainty, which is termed here the coherent region. The other region is termed the erratic region, on account of the high uncertainty in quantifying a primary orientation of spatter direction. A linear fit was applied to both the coherent and erratic regions, as annotated in Figure 6b.

**Figure 7:**
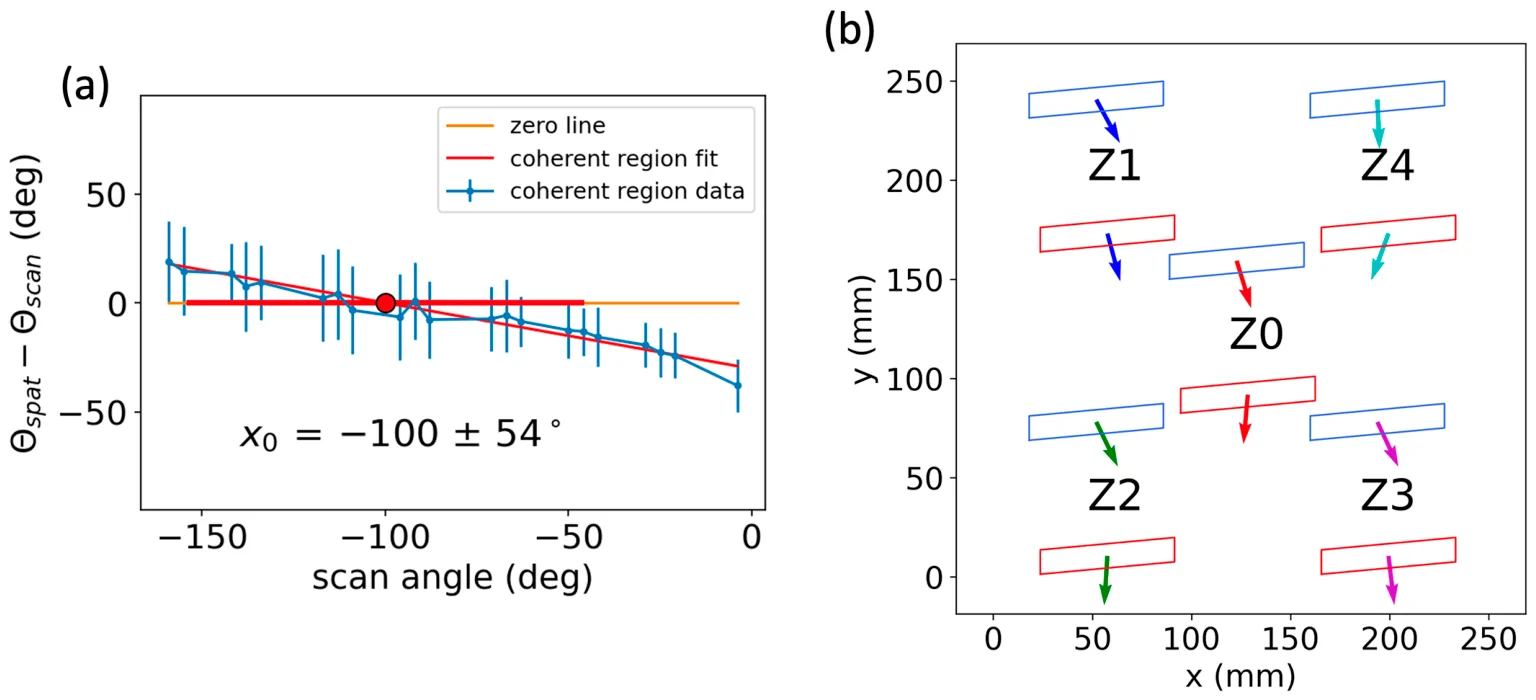
Spatter angle dependence on scan angle. (**a**) Evaluation of x-intercept for spatter–scan angle difference plot. (**b**) Direction of spatter trajectory that experiences no change in orientation, suggesting the average direction of gas flow in the ROI. Note that the orientations for the back ROIs have been rotated 180° to better agree with the estimated direction of local inert gas flow.

The x-intercept of the linear fit for the coherent region, illustrated graphically in Figure 7a, identifies the scan angle for which spatter trajectories are minimally influenced by the inert gas flow; that angle provides an estimate of the average direction of gas flow with the ROI. The direction of gas flow for each of the ROIs is presented graphically in Figure 7b, along with the outlines of each of the 10 ROIs evaluated. Note that, while the spatter observed in the back ROIs is traveling towards the back of the machine, the markers for the back ROIs point downwind (towards the front of the machine) to better represent the direction of estimated local inert gas flow. Overall, a greater degree of spatter deflection occurs in the back ROIs as evident from the greater deviation from 90°, which is expected from the headwind imposed by the gas flow.

### 4.2. Area Intensity

**Figure 8:**
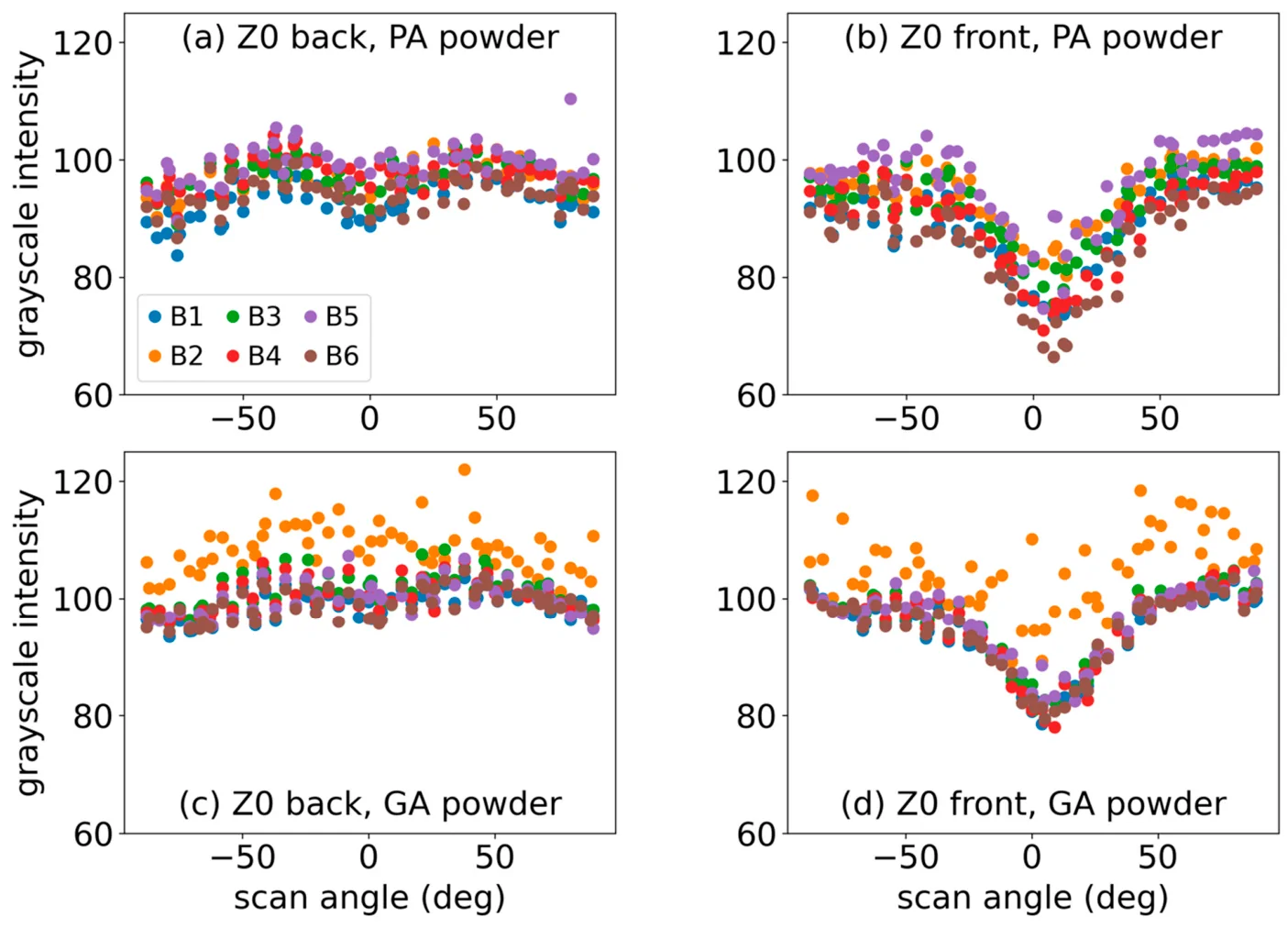
Comparison of the grayscale intensity distribution within Zone 0 for Builds 1 to 6. (**a**,**b**) show the results for the back and front ROIs, respectively, for builds performed with PA powder, and (**c**,**d**) show the results for the back and front ROIs, respectively, for builds performed with GA powder.

The average grayscale intensity distributions as a function of scan angle in the back and front ROIs of Zone 0 are shown in Figure 8a and Figure 8b, respectively; these distributions are for the builds performed using PA powder. Results for the back and front ROIs of Zone 0 for the builds performed with GA powder are shown in Figure 8c and Figure 8d, respectively. The grayscale intensity for the back ROIs exhibits a minima at 0° as well as at ±90°, and there are two maxima at ±45°. The front ROIs exhibit the minimum grayscale intensity at a scan angle near 0°, and the grayscale intensity increases to a maximum at 90°, but there are no points of interest at ±45°.

Most evident in comparing the distributions in Figure 8, the GA powder has a higher average grayscale intensity overall compared to that for the PA powder. In addition, there is more substantial scatter in the data from Build 2 of the GA powder in Figure 8c and Figure 8d with respect to the other builds. However, apart from Build 2, the grayscale intensity distribution resulting from the GA powder shows less variation over the number of builds performed. The distribution within the same ROI for the PA powder shows more random variation but still a similar trend to that exhibited by the GA powder.

**Figure 9:**
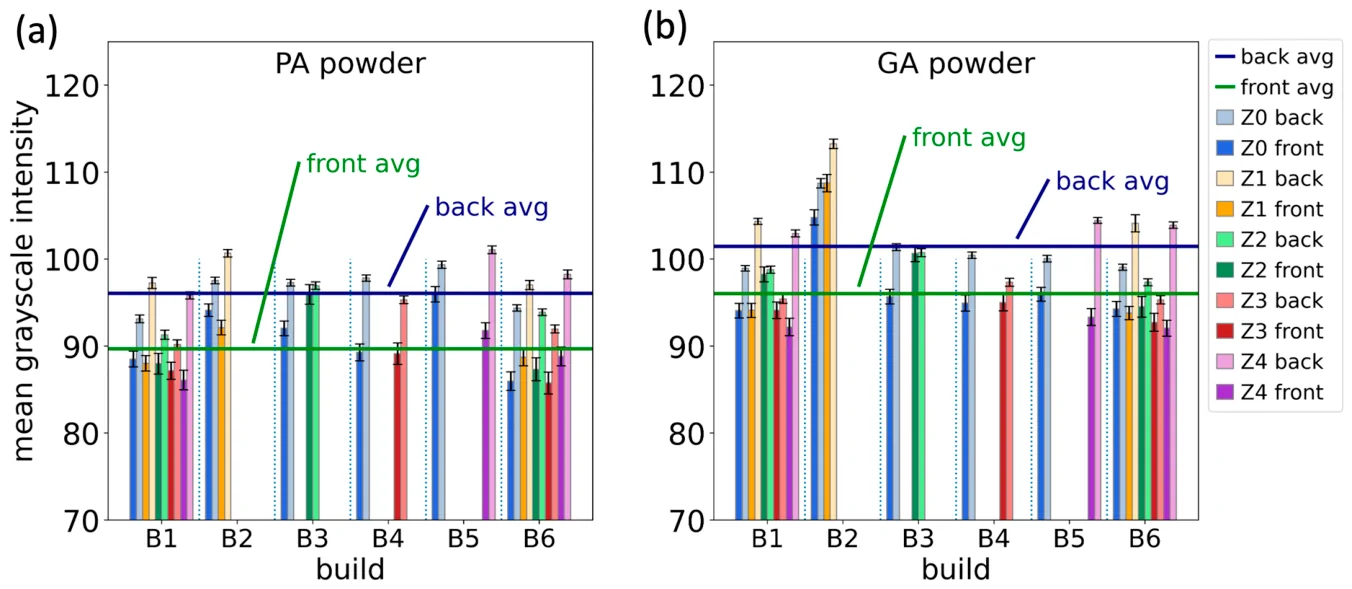
Comparison of the average inter-specimen grayscale intensity in spatter for builds performed with (**a**) PA and (**b**) GA powder. Both graphs show the average intensity for the front and back regions over the six builds.

Figure 9 compares the average inter-specimen grayscale intensity for each ROI by build, for both Figure 9a PA and Figure 9b GA powder. Through a comparison of this type, the trends in the data become more evident and the outliers can be more easily identified. For instance, the comparatively high average grayscale intensity is apparent in the results for Build 2 that was performed with the GA powder. Contrary to expectations, the average inter-specimen grayscale intensity for the back ROIs is significantly greater than that of the front ROIs in builds performed with both GA and PA powder. That quality is most clear in the inter-build intensity averages for the back and front ROIs, which are denoted by the green and blue lines, respectively. Of equal importance, the average grayscale intensities obtained for the GA powder are notably higher (by approximately 8%) than those for the PA powder. That suggests that there is a greater extent of spatter emitted during the laser–powder interaction with the GA powder.

**Figure 10:**
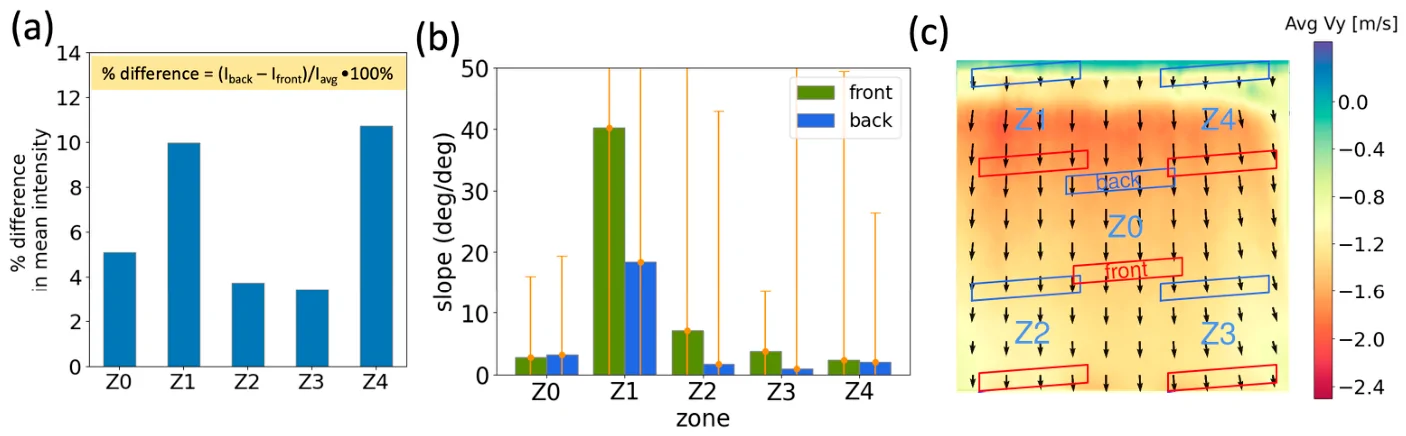
Evaluation of relationships between spatter and gas flow. Comparison of the (**a**) grayscale intensity variation in spatter (back to front) and the (**b**) slope of the erratic region of the spatter–scan angle difference plots in Figure 6b with (**c**) inert gas flow velocity distribution across the build plate (back to front) from [32] at an offset of 8 mm from the build plate. Note the high spatter difference for Zones Z1 and Z4 in (**a**), the high erratic region slope for Z1 in (**b**), and the low and high gas flow velocity in (**c**) in the Back and Front regions of the two zones of Z1, respectively.

A comparison of the difference between the grayscale intensity from the back and front inter-specimen ROIs was performed as an alternate approach to identify spatial variation across the zones. Figure 10a shows the difference in average grayscale intensity (in %) for each of the five zones for Build 1. As evident from these comparisons, the difference in average grayscale intensity between the back and front ROIs is largest in Zones 1 and 4, which are at the back of the print bed nearest the inert gas inlet grid nozzle. Reflecting on results of the spatter trajectories in Figure 10b, the slope of the erratic region of the spatter–scan angle difference plots is also greatest in Zone 1. A map of the average gas flow velocity obtained experimentally in a mock build chamber for the EOS M290 using particle image velocimetry is shown in Figure 10c [32]. The difference in the velocity of the inert gas flow between the back and front ROIs is greatest for the ROIs of Zones 1 and 4, as is the difference in grayscale intensity of spatter content from OT imaging.

### 4.3. Comparison with Tensile Properties

**Figure 11:**
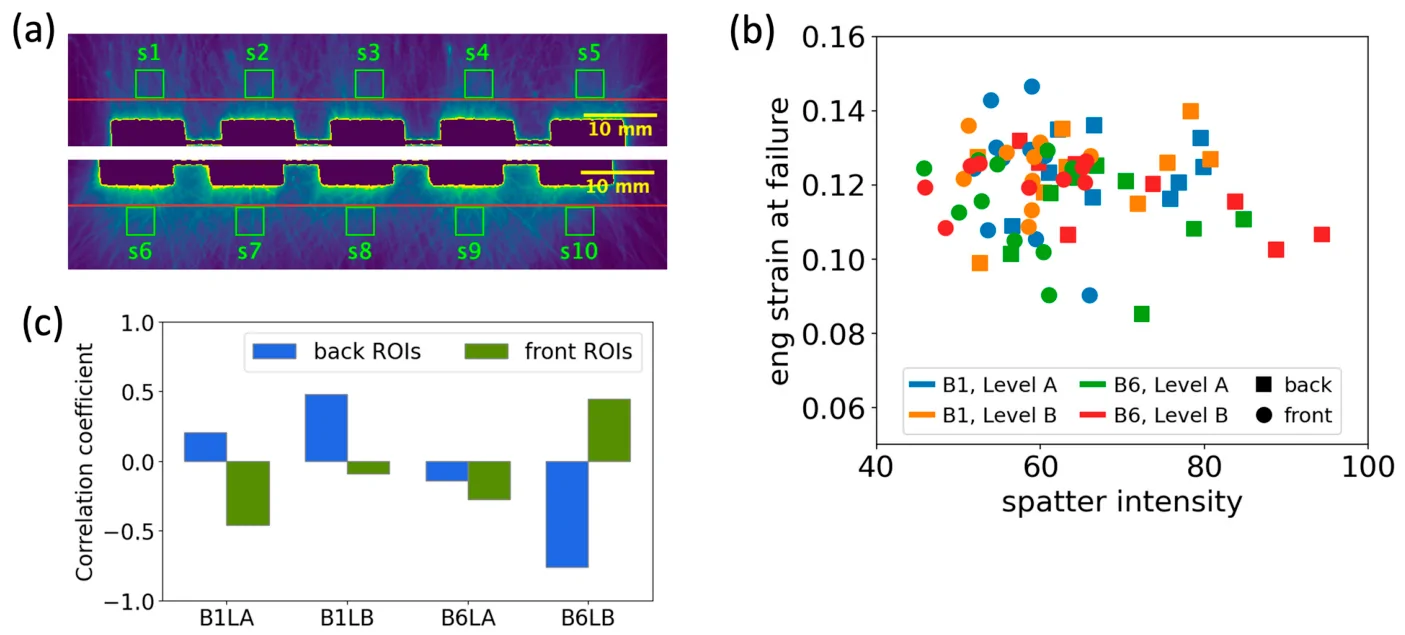
Specimen spatter regions (SSRs) used for measuring average grayscale intensity. (**a**) SSRs for back ROIs (above) and for front ROIs (below). (**b**) Comparison of specimen spatter responses in terms of the grayscale intensity of the spatter within the SSRs with engineering strain at failure for the PA powder. (**c**) Correlation coefficients for spatter–strain relationship.

To assist in comparing the spatter emitted during printing and the mechanical properties of the tensile specimens, a group of specimen spatter regions (SSRs) were defined to quantify the average grayscale intensity of spatter content adjacent to the tensile specimens, as shown in Figure 11a. There are 10 SSRs defined in this figure that correspond to the 10 vertical specimens in each zone where the SSRs s1 to s5 correspond to the back ROIs and s6 to s10 correspond to the front ROIs.

The average grayscale intensity in the SSRs was evaluated for the range of layers associated with printing the gauge section of the vertical specimens, which spans approximately 500 layers. As a result, the dataset was limited to layers for which the scan angle was within 6° of the *y*-axis (i.e., predominantly in the direction of gas flow). This restriction reduced the number of layers assessed to 30 layers, which were distributed across the specimen gauge sections. The data for the A and B level specimens was evaluated separately, for a total of about 60 layers per build.

The grayscale intensity of spatter in the SSR adjacent to each specimen was measured using an ImageJ macro. Details of this process are listed in the Supplemental Information (SI). The SSRs are shown in Figure 11a. A comparison between the grayscale intensity of spatter generated during exposure of the vertical specimens and the resulting strain at failure for those specimens is presented in Figure 11b. As spatter is indicative of printing conditions that can lead to defects, and these defects can reduce the strain at failure of printed metal, an increase in spatter content is expected to be related to a decrease in strain at failure. Grouping the specimens by build, level, and ROI, linear trends were fit to the data for spatter and strain at failure to assess potential correlations. The correlation coefficients (R values) for each fit are plotted in Figure 11c. There is an overall decrease in strain to failure with increasing spatter intensity in Figure 11b, however, there is no correlation apparent between the spatter and strain at failure of the metal.

## 5. Discussion

### 5.1. Spatter Trajectories

The trends in angular descriptions for spatter presented in Figure 6a show that spatter angle is primarily linearly related to the scan angle. This is unexpected since the motion of spatter is influenced by gravity and the force of the inert gas flow, which is non-linear. Both contribute to the curvature of the spatter paths. Admittedly, the influence of gravity is difficult to observe objectively since the OT camera is oriented vertically with regard to the powder bed rather than oriented parallel to the powder bed.

Noting that spatter particles are ejected primarily anti-parallel to the direction of laser scanning [13], and that a bi-directional scan strategy was used here, spatter is ejected bi-directionally along the orientation of laser scanning. Once the spatter particle is ejected from the melt pool it is subject to the forces of the inert gas flow, which will promote movement towards the gas outlet. The path of particles ejected upwind into the back ROIs undergoes substantial curvature, as can be observed in Figure 3a, particularly in the back of Zone 2. Conversely, particles that are ejected downwind into the front ROIs undergo much less or no curvature, since they are moving in the general direction of gas flow. For a laser scanning angle of 0°, i.e., parallel to the *x*-axis, particles ejected in the +x-direction will be deflected with angles ≤0°, while particles ejected in the -x-direction will be reoriented in a direction ≥180° (0°). Consequently, a histogram for spatter orientation including the entire range of scan angles will exhibit a bi-modal distribution. A normal Gaussian model will not fit this distribution well, which is a limitation of this approach to define the spatter angle.

The difference between the spatter and scan angles for Zone 0 is presented in Figure 6a. In response to the unique behavior in this distribution for laser scan angles near 0°, the data was redistributed in Figure 6b. The region of largest angular difference, referred to as the erratic region, is an indirect reflection of the effects from gas flow in the ROI. The erratic region reflects that gas flow is most effective at deflecting the particles from their angle of ejection for scan angles near 0°. Moreover, the slope of the data in the erratic region is highly dependent on gas velocity through drag forces imposed on the particles. For adequately high gas flow velocity, all spatter trajectories should be reoriented parallel to the direction of the gas flow, regardless of the ejection angle. In fact, the spatter paths are expected to become more coherent with increasing gas flow velocity, as well as reduce the width of the incoherent erratic region. That dependence on gas velocity should increase the amplitude of the erratic region in the spatter angle difference plots and result in a positive correlation between the gas flow rate and the slope of the erratic region. The slope of the spatter–scan angle difference plots is presented for all zones in Figure 10b and shows that the erratic region of Zone 1 was largest overall, which is located nearest the gas inlet. According to recently reported gas flow data [32,46], the region of highest gas flow velocity across the build plate occurs in the front ROI of Zone 1 as presented as a map in Figure 10c. That appears to confirm the velocity dependence.

The large uncertainty in the estimated gas flow direction in Figure 7a is at least partly attributed to the temporal variation in the gas flow [46]. It also signifies the substantial spatial fluctuations in spatter direction within the ROIs. While the positive correlation between the slope of the erratic region in the spatter–scan angle difference plots and the relative magnitude of the inert gas flow reported in [32] holds for the majority of the ROIs, it does not for the back ROI of Zone 1. The erratic region in that zone exhibits a high slope, as shown in Figure 10b, but low gas flow velocity, evident in Figure 10c. Abeyta et al. [32] reported that the gas flow in the build chamber of M290 undergoes substantial changes with respect to height above the build surface over the first 35 mm. Additionally, Soltani et al. [1] reported a stratification of powder particles by size due to the agitation experienced during the recoat cycle, resulting in a smaller size distribution of powder closer to the beginning of the recoating stroke and a larger size distribution near the end of the stroke. Laser absorptivity is related to packing condition [1] and size distribution [47]. Therefore, spatial variations in particle energetics over the powder bed could obscure the effects of gas flow. Neither of these factors has been considered in the current analysis but could contribute to variation in spatter–scan angle response.

### 5.2. Area Intensity

The inter-specimen grayscale intensity of spatter content evaluated in this investigation provides an indirect measure of the amount of spatter generated during the L-PBF process. Higher grayscale intensity observed in the temporally integrated images is representative of either a larger volume of spatter emitted or slower moving spatter particles that remain in the ROIs longer. Since larger spatter particles tend to travel slower than smaller particles [48], higher grayscale intensity in the OT images is representative of either more spatter particles or larger size; both contributions increase the total volume of spatter observed in the OT images. Due to the concern that spatter particles deflected onto the powder bed can serve as or cause the generation of metal defects [9], these measures reflect features that can contribute to build quality and reliability.

As shown in Figure 8, the inter-specimen grayscale intensity depends on the scan angle, which agrees with several authors who have identified that spatter is primarily ejected anti-parallel to the direction of the laser path [10,13,19]. In addition, the inter-specimen grayscale intensities exhibited within the back and front ROIs are unique, which is most pronounced in scanning at 0°. Comparing the spatter intensity from the back and front perspectives in Figure 8, the inter-specimen intensity at a scan angle of 0° is higher for the back ROIs relative to the front. Conversely, at a scan angle of 90° the spatter intensity is higher for the front ROIs, though the difference is less prominent. Understanding the causes requires more careful thought.

Anwar and Pham [13] observed that when spatter is ejected upwind it can re-enter the path of the laser and be remelted, which results in more spatter. However, spatter ejected downwind will not experience re-exposure as it is carried away from the melt pool by the gas flow. That is a plausible explanation for the greater spatter content observed in the back ROIs, i.e., located upwind. In addition, gas flow anomalies have been observed near the back edge of the build plate in EOS M290 [46,49], which could also contribute to spatial variation in spatter content; further investigation is necessary.

It is important to admit that the OT assessment of spatter is subject to some limitations. The off-axis mounting of the OT camera near the front of the machine results in a small projection of spatter with vertical motion in the +y-direction in the OT images. Projection effects of this imaging configuration make it appear that there is more spatter in the back ROIs than there is physically. The relative contributions of each of these two phenomena to the difference in spatter content between the back and front ROIs could be better understood by resolving the motion of spatter in 3D with plenoptic imaging [29] or high-speed imaging with two cameras [30]. Those methods were not available for this investigation, which is an admitted limitation and recommended for future work. 

If process physics in L-PBF cause an uneven distribution of spatter content, and spatter contributes to build defects [6,9,13,27,31], then the incidence of LOF defects is likely to coincide with spatter content. LOF defects can result from inadequate volumetric energy density (VED) to achieve complete fusion of the powder [9], which can happen when a large spatter particle absorbs a substantial portion of the laser energy [10,18]. Strategies for mitigating this issue could include the use of scan angles anti-parallel to the direction of gas flow, or as close to 90° as possible, such that the spatter is ejected downwind and will not be re-exposed to the laser [48]. If this is not feasible, the scan angle and/or magnitude and direction of gas flow should be selected to minimize the amount of spatter that lands on the printed regions [48].

### 5.3. Importance of Powder Type

The powder feedstock is one of the primary contributions to cost in the manufacture of components by L-PBF [50]. Due to the lower cost of the materials and processing needed, powder produced by gas atomization is an economical alternative to powder produced by plasma atomization. Moreover, the studies by Kassym et al. and Chen et al. [51,52] showed that the static strength of parts produced with GA powder is not significantly different from that of parts produced with PA powder. However, there are quality concerns with using GA powder, many of which are not fully understood. In processes where oxide contamination results in significant quality concerns, PA powders are preferred on account of the smoother and more spherical morphology compared to GA powders, as well as a lower oxide content [53]. Hatch et al. [54] evaluated the morphological properties of PA and GA powders and found that there was a higher proportion of satellites present in GA powder on the surface of the particles. The irregular morphology of the particles can interfere with establishing a uniform powder layer during the recoat cycle, contributing to process variation [54]. In addition, the apparent density for the GA powder used in this study is lower than that of the PA powder [55], suggesting reduced inter-particle contact. A lower apparent density of the powder leads to more gas between the powder particles that can be entrapped in the melt pool and cause entrained porosity. Finally, powder particles with higher surface roughness and lower flowability tend to form agglomerations of particles during recoating, which create voids around the agglomerates [1], thereby resulting in an uneven layer thickness and potentially further contributing to melt pool instability.

The builds performed with GA powder in this study exhibited greater average spatter content compared to the builds performed with PA powder as evident in Figure 9. As higher spatter content is associated with a higher incidence of defects [9], there is concern that the risk of build defects is greater with GA powder. The size distribution of the feedstock powder also has a significant effect on the build performance [1]. Hatch et al. [54] evaluated the particle size characteristics of PA and GA powders and found that there was a higher proportion of fines in the GA powder, as well as a wider size distribution overall. A powder lot with smaller average particle size has a higher absorptivity than a powder bed composed of larger particles, due to the increased number of laser ray reflections as well as reduced thermal conductivity resulting from a smaller contact area [20]. In this case, the effective incident laser power is higher in the case of GA powder, which could be responsible for the increase in spatter generated [18,19,20]. Another performance attribute influenced by the PSD is fatigue strength; Soltani et al. [1] reported superior fatigue performance of parts produced using the coarser of two size distributions of powder. Hence, the size distribution, rather than the processing method, could be the root cause. Further work on this topic related to the generation of spatter appears warranted.

Spatter can result from conditions of excessive VED, which also contributes to increased gas porosity [56]. While low levels (<2% by volume) of porosity do not have a significant influence on the static tensile properties of as-printed parts [57], the fatigue strength can be reduced significantly by that level [58,59]. Kasperovich and Hausmann [60] showed that hot isostatic pressing (HIP) treatment can improve the ductility and fatigue strength of Ti6Al4V components produced by L-PBF, but at the cost of other property changes, as well as additional time and expense. Therefore, the economy of GA powder may be advantageous if these concerns are mitigated.

### 5.4. Comparison with Tensile Properties

A relationship between the bulk spatter levels and strain to failure of the metal determined through tensile testing was not identified (Figure 11b). Of note, correlations between porosity and strain at failure are generally limited, unless the pores are on the order of 100 μm or larger [21]. As a result, the lower ductility of AM metals relative to those resulting from wrought processing is more likely due to differences in underlying microstructure than porosity [61]. Nevertheless, a relationship between porosity and the fatigue life of parts is possible. Spatial variations in powder particle size across the build space can contribute to the amount of spatter generated as well as the mechanical properties [1,61]. Spatter also exhibits a relationship with the surface roughness of parts [62], which in turn can influence the fatigue response. An understanding of the effects of spatter on the metal fatigue properties in L-PBF appears necessary and will be the focus of our future work.

### 5.5. Limitations

Results from this investigation provide new insights concerning the quality of L-PBF builds based on an analysis of spatter documented by OT in situ data. However, there are limitations to this research that must be considered. While grayscale emissions observed in the OT images are indicative of spatter content, the relationship between grayscale intensity and physical spatter content cannot be assumed to be linear. For example, the OT camera only detects radiant emissions with wavelengths within 900 ± 25 nm; spatter that has cooled significantly may not be detected. Therefore, the OT approach underestimates the total spatter emission. Furthermore, the emissivity of Ti6Al4V depends approximately linearly on temperature [63] and exhibits further variation during thermal oxidation [64]. While OT imaging is indicative of variations in spatter content, the approach in this research cannot be used to directly estimate the volume of spatter generated in a build. Methods do exist to identify the relationships between in situ signals and actual spatter content, such as collecting and measuring the spatter particles produced in order to relate to the OT images as described in [48].

Although previous research has established correlations between spatter content and OT gray values, it was performed for metal printed outside of the optimum/recommended processing window. Björkstrand et al. [65] evaluated the influence of process parameters on the spatter content observed in in situ OT imaging for a series of builds performed with 316L powder. They found that the average defect size increased substantially when the grayscale content of spatter exceeded a gray value of approximately 87 [65]. While the UW round robin study was conducted with a different material, a similar set of experiments could be designed to evaluate the gray value threshold associated with an increase in the average defect size. However, when printing with parameters within the recommended process window (parameters known to produce optimum metal quality, such as those in the present study), the reported variation in defect content was too small to establish a conclusive link with the bulk gray value [65]. As a result, while the findings of the UW round robin study have identified significant spatial variations in the grayscale levels of spatter features in OT, a quantitative connection with metal quality still needs to be developed. This limits the conclusions that can be drawn between variations in OT gray values and physical spatter content/metal quality. Nonetheless, process signatures present in OT imaging exhibit spatial features that are not captured in volumetrically aggregated grayscale statistics. Convolutional neural network (CNN) models and other computer vision methods have potential to advance efforts in distinguishing process variations indicative of defects for metal printed within the optimum processing window. That will be the focus of our future work.

In the present study [21], Build 1 was performed with virgin powder, but the powder used in each subsequent build was reused, i.e., after being filtered through a sieve to remove particles of excessive size. The variability in the condition of the powder feedstock for each build complicates comparisons between builds, as powder reuse is known to have an influence on build results [50]. Nevertheless, there was no trend in spatter content over the six builds, which suggests that the reuse process was not a factor in the volume of spatter generated in the present study. In an ideal setting, all the builds in this effort would be performed using powder from the same initial batch and powder would not have been reused between builds. If the spatter generated is a reflection of build health, there was no degradation in the powder quality over the six builds.

It was previously highlighted that the spatter trajectories are distorted due to the OT camera mount position, which is at an angle offset from the build plate normal by approximately 20°. This issue is shared with many other L-PBF systems and can be overcome by using multiple view angles to resolve the trajectories of spatter in 3D [29]. 3D spatter imaging would be a valuable addition to the in situ monitoring suites as well as an opportunity to build on this research.

In summary, this investigation has shown that in situ OT imaging is able to reveal spatial variations in the thermal features of spatter as well as the spatter kinetics and connection to the inert gas flow. Using native in situ monitoring tools already present on many AM systems, operators can both forecast and identify regions of highest concern for reduced metal quality. What remains is to establish the link between the in situ sensing modalities and quantitative measures of spatter volume, metal quality, and potential influence of powder condition. Future efforts should be focused on characterizing the dynamics of spatter in greater detail towards quantitatively forecasting metal performance.

## 6. Conclusions

In situ optical tomography (OT) data was collected during a series of L-PBF builds consisting of a distribution of tensile specimens and used to evaluate variations in spatter content. Variations in the grayscale levels of spatter features observed in OT imaging were compared for plasma atomized and gas atomized feedstock powder of Ti6Al4V and evaluated with respect to location in the build chamber. A relationship between the direction of spatter trajectories and the scan angle was applied as a means of estimating the local direction of the inert gas flow. In addition, correlations between strain at failure of the tensile specimens and the grayscale levels of spatter observed adjacent to the corresponding specimen were assessed. The grayscale levels of spatter features were found to be highest in regions closest to the inert gas nozzle at the back of the build chamber and greater for builds performed with GA powder compared to builds performed with PA powder.

## Figures and Tables

**Table 1:** Summary of research approaches made.

Author	Spatter Kinetics/3D Tracking	Processing Parameters	Melt Pool Morphology	Tensile/Quasi-Static Performance	Fatigue Performance	Microstructural Evaluation/Microscopy	Powder Characteristics	Surface Roughness	In Situ Imaging	Gas Flow Characteristics	µCT Imaging	In Situ Imaging Correlation with Characterization/Testing Data	Feedback Process Control	Process Simulation
Gunenthiram et al. [16]	X	X	X				X		X					
Wang et al. [18]	X	X	X			X	X		X					
Taheri Andani et al. [8]	X	X	X						X					
Repossini et al. [36]	X	X	X						X	X		X		
Anwar and Pham [13]		X		X		X			X	X				
Gasper et al. [12]							X							
Yin et al. [10]	X		X						X					
Dowling et al. [2]		X					X							
Khairallah et al. [19]	X	X	X				X	X	X				X	X
Chen et al. [3]		X						X						
Soltani-Tehrani et al. [1]				X	X	X	X	X			X			
Wu et al. [37]	X	X						X						
Fischer et al. [15]	X	X												
Liu and Wen [20]	X	X	X					X	X	X				X
Kelly et al. [29]	X	X				X			X					
Snow et al. [9]		X							X	X	X	X		
Snow et al. [5]		X			X	X					X			
Sunderarajan et al. [4]		X		X			X							
Montelione et al. [38]				X		X	X							
Abeyta et al. [32]		X								X				

**Table 2:** Particle size distribution for feedstock powders.

	GA Powder	PA Powder
D10 (µm)	15	30
D50 (µm)	28	41
D90 (µm)	45	57

**Table 3:** Process parameters used for printing.

Parameter	Value
Laser power (watts)	340
Scan velocity (mm/s)	1250
Layer thickness (µm)	60
Hatch spacing (µm)	120
Stripe width (mm)	5
Beam offset (µm)	100

## Data Availability

The original contributions presented in this study are included in the article. Further inquiries can be directed to the corresponding authors.
